# Tobacco Smoke Exposure Biomarker Profiles and Healthcare Utilization Patterns Among U.S. Children

**DOI:** 10.3390/toxics13110909

**Published:** 2025-10-23

**Authors:** Ashley L. Merianos, Georg E. Matt, Roman A. Jandarov, E. Melinda Mahabee-Gittens

**Affiliations:** 1 School of Human Services, University of Cincinnati, Cincinnati, OH 45221, USA; 2 Department of Psychology, Center for Tobacco and the Environment, San Diego State University, San Diego, CA 92123, USA; 3 Department of Biostatistics, Health Informatics, and Data Sciences, University of Cincinnati, Cincinnati, OH 45267, USA; 4 Division of Emergency Medicine, Cincinnati Children’s Hospital Medical Center, Cincinnati, OH 45229, USA

**Keywords:** total nicotine equivalents, tobacco-specific nitrosamines, volatile organic compounds, National Health and Nutrition Examination Survey, child

## Abstract

This study aimed to examine the associations between distinct tobacco smoke exposure (TSE) biomarkers and healthcare utilization patterns in U.S. children ages 3–11 years with and without current asthma. Secondary data from the 2013–2016 National Health and Nutrition Examination Survey were analyzed (N = 2838). TSE biomarkers included serum cotinine, urinary total nicotine equivalents (TNE2), 4-(methylnitrosamino)-1-(3-pyridyl)-1-butanol (NNAL), the NNAL/TNE2 ratio, and the N-acetyl-S-(2-cyanoethyl)-L-cysteine (2CyEMA)/TNE2 ratio. We conducted Poisson regression analyses to examine the associations between each biomarker and healthcare visits and hospitalizations within the past 12 months, adjusting for sociodemographic and home TSE covariates. Children without asthma who had higher urinary TNE2 levels (adjusted incidence rate ratio [aIRR] = 1.03, 95% confidence interval [CI] = 1.02–1.04) and children with asthma who had higher urinary 2CyEMA/TNE2 ratio levels (aIRR = 1.05, 95%CI = 1.03–1.07) were at an increased risk of having more healthcare visits. Children without asthma who had higher serum cotinine (aIRR = 1.21, 95%CI = 1.07–1.37) and higher 2CyEMA/TNE2 ratio levels (aIRR = 1.25, 95%CI = 1.14–1.37) were at an increased risk of hospitalizations. Children with asthma who had higher NNAL/TNE2 ratio levels (aIRR = 1.52, 95%CI = 1.11–2.09) were at increased risk of hospitalizations. It is important to consider comprehensive biomarkers of TSE in children, such as TNE, tobacco-specific nitrosamines, and volatile organic compounds, along with healthcare utilization patterns. Child TSE reduction policies are urgently needed.

## 1. Introduction

Although the rates of tobacco smoke exposure (TSE) among children in the U.S. have been declining over the years [1,2], approximately 38% of 3–11-year-olds remain involuntarily exposed [3]. Children can encounter tobacco smoke through secondhand exposure by breathing in both mainstream and sidestream smoke, as well as through thirdhand exposure by inhaling, ingesting, or absorbing through the skin the aged residue of secondhand smoke that lingers on surfaces, in dust, and in the air after smoking has stopped in the surrounding environment [4]. TSE, including thirdhand smoke, is a major health hazard for children compared to adults because of their physical composition (e.g., higher respiratory rates and immature detoxification pathways) and age-specific behavior (e.g., playing on the floor), which increases their contact with potentially thirdhand smoke-contaminated dust and surfaces [5]. Tobacco smoke contains at least 70 known and possible human carcinogens [6]. Negative health consequences of TSE during childhood include, but are not limited to, respiratory infections, asthma, and ear infections [7]. Specifically, TSE is a major modifiable risk factor for childhood asthma [8], and those exposed to secondhand smoke during childhood have a 32% increased risk of developing asthma [9]. Additionally, TSE has been associated with lower overall health status among children and adolescents [10,11].

TSE, measured via self-report or the widely used nicotine metabolite cotinine, has been associated with higher healthcare use among children [12,13]. However, no studies have used the optimal nicotine biomarker of total nicotine equivalents (TNE), the sum of nicotine and its metabolites in both free and glucuronide-conjugated forms (e.g., cotinine and trans-3′-hydroxycotinine), which, unlike cotinine, allows for daily estimates of nicotine intake [14]. Furthermore, nicotine metabolite measurements can underestimate exposure to tobacco-specific nitrosamines (TSNAs), including the strongest lung carcinogen, 4-(methylnitrosamino)-1-(3-pyridyl)-1-butanone (NNK), which undergoes metabolism to form 4-(methylnitrosamino)-1-(3-pyridyl)-1-butanol (NNAL) [15]. Thus, elevated NNAL levels have been linked to an increased risk of tobacco-related diseases, such as reduced lung function [16] and lung cancer [17]. Additionally, elevated NNAL levels have been associated with increased asthma exacerbations and linked to increased emergency care visits within the past 12 months among nonsmoking adults [18]. The NNAL/cotinine ratio may help distinguish thirdhand smoke from secondhand smoke exposure by providing insight into the timing of exposure because nicotine concentrations decrease within 1–3 days after TSE cessation [19], whereas NNK concentrations metabolized to NNAL in urine have a longer half-life of up to 45 days [20,21], and thus remain stable or increase over time based on its interactions with ambient oxidants [22]. Therefore, a higher NNAL/TNE ratio may reflect chronic or thirdhand smoke exposure, compared to acute or secondhand smoke exposure. Interestingly, a previous study found that a sample of children 0–9 years old who lived with smokers and had high NNAL and NNAL/cotinine ratio levels were at risk of having increased urgent care visits, but no difference was found based on cotinine levels [23]. Carcinogenic NNAL uptake is widespread in children and should be considered an important health risk [24,25].

There is a gap in the literature regarding the assessment of volatile organic compounds (VOCs) from tobacco smoke, such as acrylonitrile [26], which can be measured as N-acetyl-S-(2-cyanoethyl)-L-cysteine (2CyEMA) in the urine of nonsmokers [27]. This particular biomarker, 2CyEMA, has been linked to an increased risk of health problems, such as respiratory irritation [28], which may lead to increased healthcare utilization. This biomarker can also indicate low levels of exposure to acrylonitrile-containing products (e.g., plastics) other than tobacco combustion products [29]. Therefore, 2CyEMA normalized to TNE2 as the denominator for nicotine-related exposure in the ratio form may reduce the influence of these exposures to other non-tobacco acrylonitrile sources to better distinguish TSE patterns among children [23]. Thus, this study addresses the gap in the literature on child TSE by exploring the use of biomarkers beyond cotinine with healthcare utilization patterns among U.S. children with and without current asthma.

This study aimed to examine the associations between distinct TSE biomarkers (i.e., serum cotinine and urinary TNE2, and NNAL), biomarker ratios (i.e., NNAL/TNE2 and 2CyEMA/TNE2), and healthcare utilization patterns in a U.S. sample of children ages 3–11 years with and without current asthma. We hypothesized that children with and without current asthma who had elevated TSE biomarker levels, both individually and in ratio form, would be more likely to have increased total healthcare visits and overnight hospital stays than would children with and without asthma who had lower TSE biomarker levels.

## 2. Materials and Methods

### 2.1. Study Sample and Procedures

A secondary data analysis of the 2013–2016 National Health and Nutrition Examination Survey (NHANES) was conducted [30,31]; details of the NHANES 2013–2014 and 2015–2016 methods are available elsewhere [32,33]. This study used these two 2-year consecutive survey cycles to obtain a larger sample size for adequate statistical power. The complete laboratory data required for analysis were not available in the subsequent 2-year cycles (e.g., 2017–2018) at the time of analysis (e.g., 2CyEMA). Details of the NHANES 2013–2016 laboratory procedures and methods for collecting specimens from ≥3-year-olds are available elsewhere [34,35].

**Figure 1:**
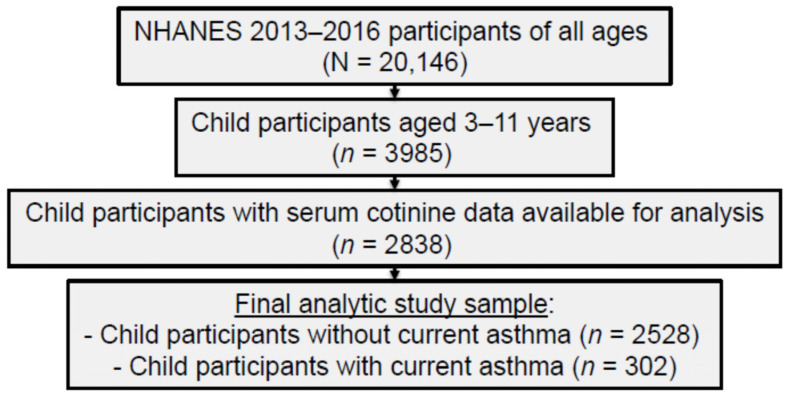
Participant flow diagram of the NHANES 2013–2016 analytic sample.

Figure 1 illustrates a participant flow diagram of the current study’s NHANES 2013–2016 analytic sample. A total of 2838 U.S. children ages 3–11 years were included after excluding those who did not have serum cotinine data (*n* = 1147). Children with (N = 2838) and without cotinine (*n* = 1147) differed based on child age (*p* < 0.001), child race and/or ethnicity (*p* < 0.001), caregiver education level (*p* < 0.001), and federal monthly poverty level (FPL; (*p* < 0.001). Children with cotinine results were older (*M* = 7.3, SD = 2.6 versus *M* = 6.3, SD = 2.53), Hispanic (28.0% versus 17.5%), had lower education of ≤high school graduate or equivalent (40.4% versus 32.6%), and had lower FPL < 185% (49.2% versus 40.2%). No differences were found based on child sex (*p* = 0.532) or home TSE status (*p* = 0.181).

Ethical review and approval for all study procedures for the NHANES 2013–2014 and 2015–2016 cycles were granted by the National Center for Health Statistics (#2011-17; 10 November 2011). Additionally, the ethical review and approval were waived by the University of Cincinnati (#2020-0350; 11 April 2020) for this secondary analysis of the NHANES 2013–2016 data with an exempt determination due to “secondary research on data or specimens (no consent required).”

### 2.2. Measures

#### 2.2.1. TSE Biomarkers

The following TSE biomarkers were assessed: (1) serum cotinine, (2) urinary TNE2, (3) urinary NNAL in individual and ratio forms with TNE2, and (4) urinary 2CyEMA in the ratio form with TNE2.

The NHANES collected data on serum cotinine levels among children aged 3 years and older in the 2013–2014 and 2015–2016 cycles. Serum cotinine, a major metabolite of nicotine, has a half-life of approximately 15–20 h [19]. Serum biomarkers were analyzed using the isotope-dilution high-performance liquid chromatography/atmospheric pressure chemical ionization tandem mass spectrometric method (HPLC-APCI MS/MS), achieving a lower limit of detection (LLOD) of 0.015 ng/mL.

NHANES collected urine samples to assess other TSE biomarkers among children aged 6 years and older in the 2013–2014 cycle and aged 3 years and older in the 2015–2016 NHANES cycle. At the time of analysis, urinary NNAL data were publicly accessible only for the 2013–2014 NHANES cycle. Urinary cotinine and hydroxycotinine levels were measured using the isotope-dilution high-performance liquid chromatography/electrospray ionization tandem mass spectrometric method (HPLC-ESI-MS/MS), achieving LLODs of 0.03 ng/mL [36,37]. TNE2, the major nicotine metabolites’ molar sum, was computed using the following formula: (total cotinine/176.2151) + (total hydroxycotinine/192.2145) nmol/mL [38,39]. Specifically, total cotinine and total hydroxycotinine included the combined free and glucuronide-conjugated forms after enzymatic hydrolysis, with the denominator referring to the molar concentrations [38,39].

Urinary NNAL, a carcinogenic NNK metabolite with a half-life of up to 45 days [20,21], was measured using isotope-dilution HPLC-ESI MS/MS, achieving an LLOD of 0.60 pg/mL [40,41]. The NNAL/TNE2 ratio was also considered in this study to potentially distinguish thirdhand smoke levels from secondhand smoke levels, because nicotine levels decrease rapidly compared to NNAL levels [4].

Urinary 2CyEMA, a VOC acrylonitrile metabolite found in tobacco smoke, among other sources in low levels (e.g., acrylic) [27,29], was measured via the ultra-performance liquid chromatography coupled with electrospray tandem mass spectrometry method (UPLC-ESI/MSMS), achieving an LLOD of 0.50 µg/L [42]. As 2CyEMA may have other sources, we delimited our analysis to assess the 2CyEMA/TNE2 ratio.

#### 2.2.2. Caregiver-Reported Healthcare Utilization

NHANES questions about healthcare utilization included (1) the number of times the child saw a healthcare professional about their health within the past 12 months (NHANES provided categories: 0, 1, 2–3, 4–5, 6–7, 8–9, 10–12, 13–15, or ≥16) and (2) whether the child had an overnight hospital stay within the past 12 months (no or yes). If the child had an overnight hospital stay, a subsequent question was asked about how many times they stayed in any hospital overnight or longer (i.e., how many hospital admissions; NHANES provided categories: 1, 2, 3, 4, 5, or ≥6).

#### 2.2.3. Current Asthma and Sociodemographic and Home TSE Covariates

NHANES included a question about whether a doctor had ever told the child that they had asthma (yes, no), followed by a follow-up question about whether they still had asthma (yes, no). Children with “yes” responses to both questions were included in the sub-sample of children with current asthma, and children with a response of “no” to both questions were included in the sub-sample of children without current asthma.

The following covariates were selected a priori: child sex (male or female), child age, child race and/or ethnicity (non-Hispanic White, Black, Other/Multiracial, or Hispanic), caregiver education level (≤high school graduate or equivalent, some college, or ≥college), FPL (<185%, 185–349%, ≥350%, or unspecified including do not know or refused), and home TSE. The reported home TSE categories were defined as follows: no home TSE or not residing with a smoker; home thirdhand smoke exposure only or residing with a smoker who refrained from smoking indoors; and home secondhand and thirdhand smoke exposure or residing with a smoker who smoked indoors within the last 7 days. Adult proxy respondents were usually parents who answered questions regarding their children’s sociodemographics and home TSE. The FPL was calculated by NHANES by creating family income to the poverty threshold ratios established by the U.S. Department of Health and Human Services, which considers geographical location and family size to assess eligibility for federal financial programs [33].

### 2.3. Statistical Analysis

Data analyses were performed using R (version 4.4.1) [43] and additional packages such as ‘survey.’ We followed the NHANES analytic guidelines and applied sampling weights to account for survey non-response and selection probability to estimate figures for U.S. children aged 3–11 years [33]. NHANES also supplied primary sampling and strata variables that were applied to the analyses for variance estimation owing to the clustered design. Prior to analysis, biomarker data were imputed using the R MissRanger package (version 2.6.1), which performed random forest-based multiple imputation with predictive mean matching. This nonparametric imputation method iteratively predicts missing values based on the observed values of other variables while preserving the multivariate distribution in the data. For values <LLOD, we included them in the imputation framework to minimize potential bias and maximize efficiency. All biomarker data underwent log transformation to transform positively skewed variables before analysis, and the geometric means (GeoMs) and 95% confidence intervals (CIs) were reported for each biomarker.

We used Poisson regression or negative binomial models, the latter of which had convergence issues owing to sparse outcome distributions. To evaluate the relationships between each biomarker measurement and healthcare visits and hospitalizations within the past 12 months among children with and without current asthma, we performed Poisson regression analyses, taking into account the covariates of the child’s age, sex, race and/or ethnicity, home TSE, caregiver’s education level, and FPL. We reported the adjusted incidence rate ratios (aIRRs) and 95%CIs.

We examined Poisson regression model diagnostics to evaluate the adequacy of each model, including deviance, Pearson χ^2^, dispersion ratios, McFadden’s pseudo-R^2^, Akaike (AIC), and Bayesian (BIC) information criteria, using the R AER Package (version 1.2-15). The deviance and Pearson χ^2^ statistics were significant in all models (*p* < 0.001), consistent with the large sample size. For the models with the total number of healthcare visits as the outcome variable of interest, McFadden’s pseudo-R^2^ was approximately 0.02, the dispersion ratios were approximately 16, and the AIC values were approximately 135,600. For the models with the total number of overnight hospital stays as the outcome variable of interest, McFadden’s pseudo-R^2^ ranged from 0.20 to 0.22, the dispersion ratios were approximately 8, and AIC values were approximately 2220. Although the dispersion ratios were >1, none of the dispersion test *p*-values were significant (all *p* > 0.987), indicating that the Poisson model was appropriate and that there was no overdispersion in these models. The residual diagnostics, deviance, and Pearson χ^2^ did not indicate undue influence from outliers or high leverage points.

## 3. Results

### 3.1. TSE Biomarker Levels in U.S. Children Ages 3–11 Years with and Without Current Asthma

Table 1 displays the descriptive statistics for the levels of child TSE biomarkers, both individually and in ratio form with TNE2, among the total child sample and by current asthma diagnosis.

### 3.2. Child Sociodemographic and Home TSE Characteristics

Table 2 presents the sociodemographic and home TSE characteristics of the subsamples of children with and without current asthma. The average (SD) age of the children was 7.3 (2.6) years, and 49.0% of the children were girls. Nearly half of the children were non-Hispanic White (48.1%), followed by 28.0% Hispanic, 13.7% non-Hispanic Black, and 10.2% of non-Hispanic Other race or multiracial background. Education level varied among caregivers, with 27.6% having at least graduated from college, and approximately half of the households had the lowest FPL < 185% (49.2%). Concerning child home TSE, 16.1% of children had home thirdhand smoke exposure only or resided with smokers who smoked outdoors only, and 8.0% of children had home secondhand and thirdhand smoke exposure or resided with smokers who smoked indoors (see Table 2). 

### 3.3. Child TSE Biomarker Levels Based on Total Number of Healthcare Visits Within 12 Months

**Figure 2:**
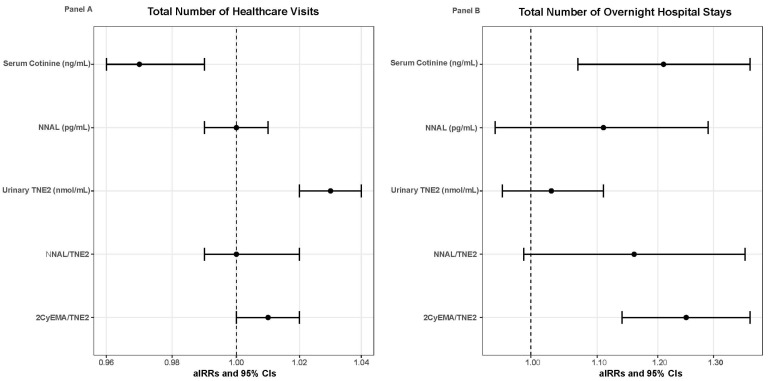
Forest box plots illustrating the results from the Poisson regression models for children without current asthma, with the dots as the aIRRs and the horizontal lines as the 95 %CIs. Panel (**A**) illustrates the associations between TSE biomarkers and the total number of healthcare visits. Panel (**B**) illustrates the associations between TSE biomarkers and the total number of overnight hospital stays.

Overall, children without current asthma averaged 1.87 (SE = 0.03) healthcare visits within the past 12 months. Children without current asthma who had higher urinary TNE2 levels (aIRR = 1.03, 95%CI = 1.02–1.04) were at an increased risk of having more healthcare visits within the past 12 months (ꭓ^2^ = 35,566.55, df = 2275, *p* < 0.001) (Table 3). Conversely, children without current asthma and with elevated serum cotinine levels (aIRR = 0.97, 95%CI = 0.96–0.99) were at a decreased risk of having more healthcare visits within the past 12 months (ꭓ^2^ = 35,669.78, df = 2275, *p* < 0.001). No differences were found between the urinary biomarkers of NNAL (ꭓ^2^ = 35,654.63, df = 2275, *p* < 0.001), NNAL/TNE2 ratio (ꭓ^2^ = 35,643.32, df = 2275, *p* < 0.001), or 2CyEMA/TNE2 ratio levels (ꭓ^2^ = 35,636.37, df = 2275, *p* < 0.001) with the total number of healthcare visits within the past 12 months among children without current asthma. Figure 2 Panel A displays the forest plots of the associations between the TSE biomarkers and the total number of healthcare visits among children without current asthma.

**Figure 3:**
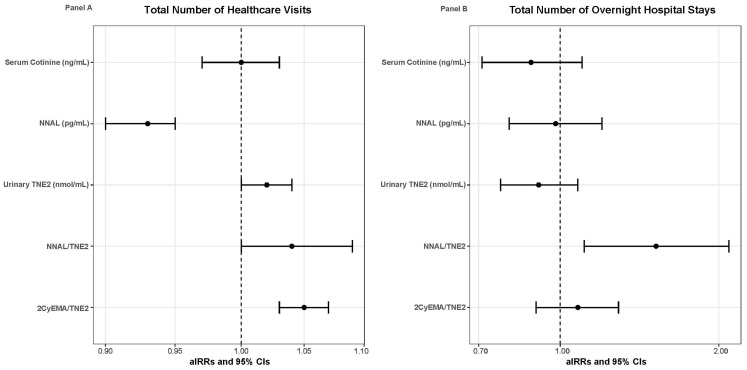
Forest box plots illustrating the results from the Poisson regression models for children with current asthma, with the dots as the aIRRs and the horizontal lines as the 95% CIs. Panel (**A**) illustrates the associations between TSE biomarkers and the total number of healthcare visits. Panel (**B**) illustrates the associations between TSE biomarkers and the total number of overnight hospital stays.

Overall, children with current asthma averaged 3.15 (SE = 0.11) healthcare visits within the past 12 months. Children with current asthma who had higher urinary 2CyEMA/TNE2 ratio levels (aIRR = 1.05, 95%CI = 1.03–1.07) were at increased risk of having more healthcare visits within the past 12 months (ꭓ^2^ = 4295.95, df = 259, *p* < 0.001) (see Table 3). Conversely, children with current asthma who had higher NNAL levels (aIRR = 0.93, 95%CI = 0.90–0.95) were at a decreased risk of having more healthcare visits within the past 12 months (ꭓ^2^ = 4252.71, df = 259, *p* < 0.001). No differences were found between serum cotinine (ꭓ^2^ = 4290.76, df = 259, *p* < 0.001), urinary TNE2 (ꭓ^2^ = 4277.92, df = 259, *p* < 0.001), and NNAL/TNE2 ratio levels (ꭓ^2^ = 4299.22, df = 259, *p* < 0.001) with the total number of healthcare visits within the past 12 months among children with current asthma. Figure 3 Panel A displays the forest plots of the associations between the TSE biomarkers and the total number of healthcare visits among children with current asthma.

### 3.4. Child TSE Biomarker Levels Based on Total Number of Overnight Hospital Stays Within 12 Months

Overall, children without current asthma averaged 1.37 (SE = 0.02) overnight hospital stays within the past 12 months. Children with higher serum cotinine levels (aIRR = 1.21, 95%CI = 1.07–1.37; ꭓ^2^ = 339.46, df = 36, *p* < 0.001) and higher 2CyEMA/TNE2 ratio levels (aIRR = 1.25, 95%CI = 1.14–1.37; ꭓ^2^ = 349.56, df = 36, *p* < 0.001) were at increased risk of having more overnight hospital stays within the past 12 months (see Table 3). No differences were found between the urinary biomarkers of TNE2 (ꭓ^2^ = 366.93, df = 36, *p* < 0.001), NNAL (ꭓ^2^ = 359.67, df = 36, *p* < 0.001), or NNAL/TNE2 ratio levels (ꭓ^2^ = 370.63, df = 36, *p* < 0.001) with the total number of overnight hospital stays within the past 12 months among children without current asthma. Figure 2 Panel B displays the forest plots of the associations between the TSE biomarkers and the total number of overnight hospital stays among children without current asthma.

Overall, children with current asthma averaged 1.51 (SE = 0.19) overnight hospital stays within the past 12 months. Children with current asthma who had higher NNAL/TNE2 ratio levels (aIRR = 1.52, 95%CI = 1.11–2.09) were at increased risk of having more overnight hospital stays within the past 12 months (ꭓ^2^ = 67.78, df = 8, *p* < 0.001). No differences were found between the other TSE biomarkers of serum cotinine (ꭓ^2^ = 74.84, df = 8, *p* < 0.001), urinary TNE2 (ꭓ^2^ = 72.48, df = 8, *p* < 0.001), urinary NNAL (ꭓ^2^ = 73.71, df = 8, *p* < 0.001), and urinary 2CyEMA/TNE2 ratio levels (ꭓ^2^ = 72.48, df = 8, *p* < 0.001) with the total number of overnight hospital stays within the past 12 months among children with current asthma. Figure 3 Panel B displays the forest plots of the associations between the TSE biomarkers and the total number of overnight hospital stays among children with current asthma.

## 4. Discussion

The present study examined the relationship between specific TSE biomarkers and patterns of healthcare use among children aged 3–11 years with and without current asthma in the U.S. As hypothesized, children without current asthma who had higher urinary TNE2 levels faced a greater risk of having an increased total number of healthcare visits within the past 12 months after controlling for the important child and family covariates of children’s sex, age, race and/or ethnicity, home TSE status, caregiver education, and FPL. Although specific information about healthcare visits was not obtained from the NHANES 2013–2016, prior research indicates that children exposed to TSE pollutants have an increased likelihood of higher healthcare resource utilization patterns, such as laboratory testing and receiving medications during healthcare visits [44]. However, the results of the current study also indicate that children without current asthma who had higher serum cotinine levels were at a decreased risk of having more healthcare visits within the past 12 months. Prior NHANES 2007–2012 research also indicated that adolescents with low and high cotinine levels were less likely to have had a healthcare visit within the past 12 months [45]. One explanation for the varied findings of serum cotinine and urinary TNE2 in this study is that, while urinary cotinine is factored into the TNE2 sum to allow for nicotine daily intake estimates, there is less influence from metabolic patterns on TNE2 than on cotinine [14]. Another explanation for this counterintuitive finding is that serum cotinine measures acute nicotine exposure, which can include intermittent exposure that may not manifest as clinically relevant, whereas TNE2 measures more cumulative and chronic nicotine exposure that may manifest symptoms and prompt more frequent healthcare visits among U.S. children without current asthma.

In addition to the nicotine biomarkers of cotinine and TNE2, we also assessed the VOC acrylonitrile metabolite 2CyEMA, which is present in tobacco smoke and other sources such as acrylic, and thus normalized to TNE2 in ratio form in this study [27,29]. We report that children with current asthma who had higher 2CyEMA/TNE2 ratio levels had a higher risk of having an increased total number of healthcare visits within the past 12 months after adjusting for important child and family covariates. Additionally, children without current asthma who had higher serum cotinine and urinary 2CyEMA/TNE2 ratio levels were at an increased risk of having more overnight hospital stays within the past 12 months. These results are especially concerning since children without current asthma who had higher serum cotinine and urinary 2CyEMA/TNE2 ratio levels had a 21% and 25% excess risk, respectively, of having more overnight hospitalizations, even after considering important child and family covariates, including home TSE status. Our findings of higher serum cotinine levels and overnight hospital stays align with prior NHANES 2009–2012 research that also found that higher cotinine levels increased the risk of having more overnight hospital stays among 3–19-year-olds [12]. Additionally, prior research on children 0–9 years old who were clinically ill and lived with smokers found that for every one-unit increase in salivary cotinine, children were at a 50% excess risk for hospital admissions over 6 months [13]. Pediatric tobacco control efforts initiated in inpatient settings are encouraged to decrease TSE-associated morbidity in hospitalized children.

Concerning the urinary TSNA findings, the current study found that children with current asthma who had higher NNAL/TNE2 ratio levels were at increased risk of having more overnight hospital stays, but those with current asthma who had higher NNAL levels were at decreased risk of having more healthcare visits within the past 12 months. However, we found no association between urinary NNAL or NNAL/TNE2 ratio levels and the total number of healthcare visits or overnight hospital stays within the past 12 months among children without current asthma. This is interesting because NNAL has a long half-life that can be sensitive to sporadic exposure and is detected 6–12 weeks after cessation of TSE [20], and has a lengthy half-life of up to 45 days [14]. However, this aligns with prior research that assessed urinary cotinine and NNAL in individual and ratio forms among children who lived with smokers and reported no differences between these biomarkers and total hospital admissions over 6 months [23]. Longitudinal research is needed to further elucidate the relationship between these TSE biomarkers and hospitalizations in children with and without current asthma.

This study has several strengths, including using NHANES 2013–2016 biomarker and self-reported data, with findings generalizable to U.S. children 3–11 years old. However, this study also had limitations. The NHANES is cross-sectional, and causal or longitudinal associations cannot be examined. We could not establish temporal relationships between child TSE and healthcare utilization outcomes. We also identified sociodemographic differences based on our study criteria of including children with cotinine data compared to excluding children without cotinine data, suggesting a potential selection bias due to exclusion. Another potential limitation is that we used caregiver-reported current asthma and did not have access to children’s medical records or clinical assessments to verify the diagnoses, potentially introducing misclassification due to reporting or recall bias. Although we used robust NHANES 2013–2016 data, information on exposure to specific tobacco products was not collected (e.g., e-cigarettes and combustible cigarettes). While NHANES data have shown that serum cotinine levels have remained from 2.20 ng/mL in 2013–2014 to 2.10 ng/mL in 2017–2020 among U.S. children [46], future studies using more recent data from 2021 to present are suggested to assess whether the current study’s associations persist. Furthermore, NHANES does not collect specific information about the types of healthcare visits (e.g., primary care and subspecialties), reasons for these visits (e.g., asthma exacerbation), and overnight hospital stays (e.g., illness versus injury). Due to the use of these secondary data, we were unable to confirm past 12 month healthcare visits and overnight hospital stays with medical records, which may have led to caregiver reporting or recall bias, especially when asking about a 12 month time frame. Despite these limitations, the two measures of total healthcare visits and overnight hospital stays within 12 months potentially capture the overall health burden of TSE across multiple health conditions and healthcare settings. 

Furthermore, the NHANES collected self-reported primary tobacco product use among ≥12-year-olds. Thus, we were unable to apply the exclusion criteria for primary tobacco product use in <12-year-olds. Only one subsample of children involved in the NHANES 2013–2016 had urinary data available for analysis, which still provided a unique opportunity to assess biomarkers rarely available for children at the U.S. population level. NHANES randomly selected this child subsample, and we applied imputation methods and weights to ensure statistical power and to generate results that are generalizable to the U.S. child population despite the smaller sample size. Longitudinal studies using more recent data are needed to better understand the relationship between distinct TSE biomarkers, which have various half-lives, and healthcare utilization patterns among U.S. children with and without current asthma.

## 5. Conclusions

This study fills a gap in the current literature by using comprehensive biomarkers to examine the link between TSE and healthcare utilization patterns among U.S. children with and without current asthma, above and beyond important covariates. U.S. children without current asthma who had higher serum cotinine levels were at an increased risk of having a higher frequency of overnight hospitalizations but were at a decreased risk of having a higher frequency of total healthcare visits. Furthermore, children without current asthma who had higher TNE2 levels, a more stable measure of daily nicotine intake than cotinine [14], had a higher risk of frequent healthcare visits. The VOC/nicotine metabolite ratio of 2CyEMA/TNE2 increased the risk of having a higher frequency of overnight hospital stays among U.S. children without current asthma. The findings in children with current asthma varied. Specifically, children with current asthma who had higher 2CyEMA/TNE2 ratio levels were at increased risk of having a higher frequency of healthcare visits, whereas those with higher NNAL levels were at a decreased risk of having a higher frequency of healthcare visits. Children with current asthma and higher NNAL/TNE2 ratio levels had a higher frequency of overnight hospital stays.

In summary, to accurately represent objectively measured TSE in children, it is crucial to consider various exposure biomarkers. Relying solely on child cotinine, which is commonly reported in the literature, may not provide a complete picture of TSE patterns in children with and without current asthma. Incorporating systematic TSE screening into healthcare visits and hospital admission protocols could assist healthcare providers in identifying at-risk pediatric patients for exposure and may provide a unique opportunity to counsel and/or refer their family members to tobacco cessation resources. While universal biomarker screening may not be feasible in routine healthcare, biomarker screening for at-risk populations, such as hospitalized children with asthma, may serve as a valuable tool for validating TSE. Future longitudinal research is needed to better understand the intricate associations between TSE and healthcare visits among children with and without current asthma to inform TSE policies and preventive and control measures. TSE reduction interventions and policies are critically needed to reduce TSE-related harm and potentially decrease associated healthcare utilization patterns among children.

## Figures and Tables

**Table 1:** Descriptive Statistics for Child TSE Biomarkers, NHANES 2013–2016.

		Overall (N = 2838)	No Current Asthma ((*n* = 2528)	Current Asthma ((*n* = 302)
Biomarker Variable	*n* (Imputed %) ^a^	GeoM (95% CI) ^b^	GeoM (95% CI) ^b^	GeoM (95% CI) ^b^
Serum Nicotine Metabolite				
Serum Cotinine (ng/mL)	2838 (0.0)	0.05 (0.04, 0.05)	0.05 (0.04, 0.05)	0.08 (0.06, 0.10)
Urinary Nicotine Metabolite				
Urinary TNE2 (nmol/mL)	919 (68.0)	0.01 (0.01, 0.01)	0.01 (0.01, 0.01)	0.01 (0.01, 0.01)
Urinary TSNA				
NNAL (pg/mL)	1001 (65.4)	1.63 (1.54, 1.73)	1.53 (1.45, 1.62)	2.32 (1.92, 2.8)
NNAL/TNE2	301 (89.5)	169.76 (162.35, 177.51)	179.46 (170.93, 188.42)	132.04 (120.11, 145.16)
Urinary VOC				
2CyEMA/TNE2	913 (68.3)	129.21 (120.9, 138.08)	131.05 (122.33, 140.40)	91.64 (74.48, 112.75)

Abbreviations: TSE, tobacco smoke exposure; NHANES, National Health and Nutrition Examination Survey; GeoM, geometric mean; CI, confidence interval; TNE2, total nicotine equivalents 2; TSNA, tobacco-specific nitrosamine; NNAL, 4-(methylnitrosamino)-1-(3-pyridyl)-1-butanol; VOC, volatile organic compound; 2CyEMA, N-acetyl-S-(2-cyanoethyl)-L-cysteine. ^a^ *n* (%) refers to the unweighted count and weighted row percentage of the imputed values, which were imputed using random forest-based multiple imputation with predictive mean matching. ^b^ GeoM and 95% CI refer to weighted imputed values.

**Table 2:** Child Sociodemographic and Home TSE Characteristics, NHANES 2013–2016.

	Overall (N = 2838)	No Current Asthma (*n* = 2528)	Current Asthma (*n* = 302)
Characteristic	*n* (%) ^a^	*n* (%) ^a^	*n* (%) ^a^
Child Age, *M* (SD)	7.3 (2.6)	7.2 (2.5)	7.7 (2.6)
Child Sex			
Male	1455 (51.0)	1270 (50.1)	181 (59.3)
Female	1383 (49.0)	1258 (49.9)	121 (40.7)
Child Race and/or Ethnicity			
Non-Hispanic White	717 (48.1)	648 (48.6)	68 (43.1)
Non-Hispanic Black	681 (13.7)	582 (13.1)	95 (19.3)
Non-Hispanic Other/Multiracial	410 (10.2)	367 (10.2)	43 (10.9)
Hispanic	1030 (28.0)	931 (28.1)	96 (26.7)
Caregiver Education Level			
≤High school graduate/equivalent	1300 (40.4)	1169 (40.7)	127 (37.0)
Some college	879 (32.0)	768 (31.3)	109 (38.5)
≥College graduate	571 (27.6)	517 (28.0)	52 (24.5)
FPL			
<185%	1608 (49.2)	1427 (48.8)	178 (52.9)
185–349%	539 (24.6)	479 (24.8)	59 (22.9)
≥350%	478 (25.7)	430 (25.9)	47 (24.2)
Unspecified	9 (0.5)	9 (0.5)	0 (0)
Child Home TSE Status			
No home TSE	2087 (75.9)	1879 (76.5)	203 (70.9)
Home thirdhand smoke exposure only	450 (16.1)	382 (15.6)	65 (19.9)
Home secondhand and thirdhand smoke exposure	261 (8.0)	230 (7.9)	31 (9.2)

Abbreviations: TSE, tobacco smoke exposure; NHANES, National Health and Nutrition Examination Survey; FPL, federal poverty level. ^a^ *n* (%) refers to the unweighted count and weighted column percentage, unless otherwise noted. Missing values were excluded.

**Table 3:** Child TSE Biomarkers by Total Number of Healthcare Visits and Overnight Hospital Stays Within the Past 12 Months, NHANES 2013–2016.

	Adjusted GeoM (95% CI)	Total Healthcare Visits	*p*-Value	Adjusted GeoM (95% CI)	Total Overnight Hospital Stays	*p*-Value
		aIRR (95% CI) ^a^			aIRR (95% CI) ^a^	
No Current Asthma						
Serum Nicotine Metabolite						
Serum Cotinine (ng/mL)	0.37 (0.34, 0.40)	0.97 (0.96, 0.99)	<0.001	0.33 (0.22, 0.50)	1.21 (1.07, 1.37)	0.002
Urinary Nicotine Metabolite						
Urinary TNE2 (nmol/mL)	0.15 (0.13, 0.17)	1.03 (1.02, 1.04)	<0.001	0.23 (0.11, 0.46)	1.03 (0.96, 1.11)	0.369
Urinary TSNA						
NNAL (pg/mL)	1.38 (1.25, 1.52)	1.00 (0.99, 1.01)	0.916	1.13 (0.81, 1.59)	1.11 (0.95, 1.29)	0.189
NNAL/TNE2	9.51 (8.72, 10.37)	1.00 (0.99, 1.02)	0.470	8.71 (6.40, 11.84)	1.16 (0.99, 1.36)	0.075
Urinary VOC						
2CyEMA/TNE2	7.41 (6.58, 8.34)	1.01 (1.00, 1.02)	0.064	5.46 (3.20, 9.30)	1.25 (1.14,1.37)	<0.001
Current Asthma						
Serum Nicotine Metabolite						
Serum Cotinine (ng/mL)	0.43 (0.39, 0.48)	1.00 (0.97, 1.03)	0.897	-	0.88 (0.71, 1.10)	0.277
Urinary Nicotine Metabolite						
Urinary TNE2 (nmol/mL)	0.14 (0.12, 0.16)	1.02 (1.00, 1.04)	0.050	-	0.91 (0.77, 1.08)	0.279
Urinary TSNA						
NNAL (pg/mL)	1.63 (1.41, 1.88)	0.93 (0.90, 0.95)	<0.001	-	0.98 (0.80, 1.20)	0.845
NNAL/TNE2	8.23 (7.63, 8.87)	1.04 (1.00, 1.09)	0.069	-	1.52 (1.11, 2.09)	0.009
Urinary VOC						
2CyEMA/TNE2	6.64 (5.66, 7.79)	1.05 (1.03, 1.07)	<0.001	-	1.08 (0.90, 1.29)	0.408

Abbreviations: TSE, tobacco smoke exposure; NHANES, National Health and Nutrition Examination Survey; aIRR, adjusted incident rate ratio; GeoM, geometric mean; CI, confidence interval; TNE2, total nicotine equivalents 2; TSNA, tobacco-specific nitrosamine; NNAL, 4-(methylnitrosamino)-1-(3-pyridyl)-1-butanol; VOC, volatile organic compound; 2CyEMA, N-acetyl-S-(2-cyanoethyl)-L-cysteine. ^a^ Poisson regression results adjusted for child age, sex, race and/or ethnicity, caregiver education level, federal poverty level, and home TSE status.

## Data Availability

The data analyzed in this study were obtained from the National Center for Health Statistics at https://wwwn.cdc.gov/nchs/nhanes/ (accessed on 1 September 2020).

## Abbreviations

The following abbreviations are used in this manuscript:

2CyEMA	N-acetyl-S-(2-cyanoethyl)-L-cysteine
aIRR	adjusted incidence rate ratio
CI	confidence interval
GeoM	geometric mean
NHANES	National Health and Nutrition Examination Survey
NNAL	4-(methylnitrosamino)-1-(3-pyridyl)-1-butanol
TNE2	total nicotine equivalents
TSE	tobacco smoke exposure
TSNA	tobacco-specific nitrosamine
VOC	volatile organic compound

## References

[1] Merianos A.L., Jandarov R.A., Choi K., Mahabee-Gittens E.M. (2019). Tobacco smoke exposure disparities persist in U.S. children: NHANES 1999–2014. Prev. Med..

[2] Tsai J., Homa D.M., Gentzke A.S., Mahoney M., Sharapova S.R., Sosnoff C.S., Caron K.T., Wang L., Melstrom P.C., Trivers K.F. (2018). Exposure to secondhand smoke among nonsmokers—United States, 1988–2014. MMWR. Morb. Mortal. Wkly. Rep..

[3] Brody D.J., Lu Z., Tsai J. (2019). Secondhand smoke exposure among nonsmoking youth: United States, 2013–2016. NCHS Data Brief.

[4] Jacob P., Benowitz N.L., Destaillats H., Gundel L., Hang B., Martins-Green M., Matt G.E., Quintana P., Samet J.M., Schick S.F. (2017). Thirdhand smoke: New evidence, challenges, and future directions. Chem. Res. Toxicol..

[5] World Health Organization (2011). Summary of Principles for Evaluating Health Risks in Children Associated with Exposure to Chemicals.

[6] International Agency for Research on Cancer Working Group on the Evaluation of Carcinogenic Risks to Humans (2004). Tobacco Smoke and Involuntary Smoking. IARC Monographs on the Evaluation of Carcinogenic Risks to Humans.

[7] U.S. Department of Health and Human Services (2014). The Health Consequences of Smoking—50 Years of Progress. A Report of the Surgeon General.

[8] Abreo A., Gebretsadik T., Stone C.A., Hartert T.V. (2018). The impact of modifiable risk factor reduction on childhood asthma development. Clin. Transl. Med..

[9] Tinuoye O., Pell J.P., Mackay D.F. (2013). Meta-analysis of the association between secondhand smoke exposure and physician-diagnosed childhood asthma. Nicotine Tob. Res..

[10] Heshmat R., Qorbani M., Safiri S., Eslami-Shahr Babaki A., Matin N., Motamed-Gorji N., Motlagh M.-E., Djalalinia S., Ardalan G., Mansourian M. (2017). Association of passive and active smoking with self-rated health and life satisfaction in Iranian children and adolescents: The CASPIAN IV study. BMJ Open.

[11] Merianos A.L., Jandarov R.A., Mahabee-Gittens E.M. (2018). Adolescent tobacco smoke exposure, respiratory symptoms, and emergency department use. Pediatrics.

[12] Merianos A.L., Jandarov R.A., Mahabee-Gittens E.M. (2017). Secondhand smoke exposure and pediatric healthcare visits and hospitalizations. Am. J. Prev. Med..

[13] Merianos A.L., Jandarov R.A., Mahabee-Gittens E.M. (2021). High cotinine and healthcare utilization disparities among low-income children. Am. J. Prev. Med..

[14] Benowitz N.L., Bernert J.T., Foulds J., Hecht S.S., Jacob III P., Jarvis M.J., Joseph A., Oncken C., Piper M.E. (2019). Biochemical verification of tobacco use and abstinence: 2019 update. Nicotine Tob. Res..

[15] Benowitz N., Goniewicz M.L., Eisner M.D., Lazcano-Ponce E., Zielinska-Danch W., Koszowski B., Sobczak A., Havel C., Jacob III P. (2010). Urine cotinine underestimates exposure to the tobacco-derived lung carcinogen 4-(methylnitrosamino)-1-(3-pyridyl)-1-butanone in passive compared with active smokers. Cancer Epidemiol. Biomarkers Prev..

[16] Lei X., Wen H., Xu Z. (2023). Relationship between urinary tobacco-specific nitrosamine 4-(methylnitrosamino)-1-(3-pyridyl)-1-butanol (NNAL) and lung function: Evidence from NHANES 2007–2012. Tob. Induc. Dis..

[17] Park E.Y., Lim M.K., Park E., Oh J.-K., Lee D.-H. (2021). Relationship between urinary 4-(methylnitrosamino)-1-(3-pyridyl)-1-butanol and lung cancer risk in the general population: A community-based prospective cohort study. Front. Oncol..

[18] Ho G., Tang H., Robbins J.A., Tong E.K. (2013). Biomarkers of tobacco smoke exposure and asthma severity in adults. Am. J. Prev. Med..

[19] Tobacco and Volatiles Branch (2017). Laboratory Procedures Manual: Cotinine and Hydroxycotinine—Serum and Saliva.

[20] Goniewicz M.L., Havel C.M., Peng M.W., Jacob III P., Dempsey D., Yu L., Zielinska-Danch W., Koszowski B., Czogala J., Sobczak A. (2009). Elimination kinetics of the tobacco-specific biomarker and lung carcinogen 4-(methylnitrosamino)-1-(3-pyridyl)-1-butanol. Cancer Epidemiol. Biomarkers Prev..

[21] Hecht S.S., Stepanov I., Carmella S.G. (2016). Exposure and metabolic activation biomarkers of carcinogenic tobacco-specific nitrosamines. Acc. Chem. Res..

[22] Schick S.F., Glantz S. (2007). Concentrations of the carcinogen 4-(methylnitrosamino)-1-(3-pyridyl)-1-butanone in sidestream cigarette smoke increase after release into indoor air: Results from unpublished tobacco industry research. Cancer Epidemiol. Biomarkers Prev..

[23] Merianos A.L., Jandarov R.A., Mahabee-Gittens E.M. (2023). Carcinogenic and tobacco smoke-derived particulate matter biomarker uptake and associated healthcare patterns among children. Pediatr. Res..

[24] Hecht S.S., Ye M., Carmella S.G., Fredrickson A., Adgate J.L., Greaves I.A., Church T.R., Ryan A.D., Mongin S.J., Sexton K. (2001). Metabolites of a tobacco-specific lung carcinogen in the urine of elementary school-aged children. Cancer Epidemiol. Biomarkers Prev..

[25] Stepanov I., Hecht S.S., Duca G., Mardari I. (2006). Uptake of the tobacco-specific lung carcinogen 4-(methylnitrosamino)-1-(3-pyridyl)-1-butanone by Moldovan children. Cancer Epidemiol. Biomarkers Prev..

[26] St Helen G., Jacob III P., Peng M., Dempsey D.A., Hammond S.K., Benowitz N.L. (2014). Intake of toxic and carcinogenic volatile organic compounds from secondhand smoke in motor vehicles. Cancer Epidemiol. Biomarkers Prev..

[27] Bhandari D., Zhang L., Zhu W., De Jesús V.R., Blount B.C. (2021). Optimal cutoff concentration of urinary cyanoethyl mercapturic acid for differentiating cigarette smokers from nonsmokers. Nicotine Tob. Res..

[28] Agency for Toxic Substances and Disease Registry (2025). Toxicological Profile for Acrylonitrile.

[29] International Agency for Research on Cancer (1999). Re-evaluation of some organic chemicals, hydrazine and hydrogen peroxide. Proceedings of the IARC Working Group on the Evaluation of Carcinogenic Risks to Humans. International Agency for Research on Cancer Press: Lyon, France, 1998. IARC Monogr. Eval. Carcinog. Risks Hum..

[30] Centers for Disease Control and Prevention, National Center for Health Statistics (2014). National Health and Nutrition Examination Survey Data.

[31] Centers for Disease Control and Prevention, National Center for Health Statistics (2016). National Health and Nutrition Examination Survey Data.

[32] Johnson C.L., Dohrmann S.M., Burt V.L., Mohadjer L.K. (2014). National Health and Nutrition Examination Survey: Sample design, 2011–2014. Vital Health Stat. 2.

[33] Centers for Disease Control and Prevention (2018). National Health and Nutrition Examination Survey: Analytic Guidelines, 2011–2014 and 2015–2016.

[34] Centers for Disease Control and Prevention (2013). National Health and Nutrition Examination Survey (NHANES): MEC Laboratory Procedures Manual.

[35] Centers for Disease Control and Prevention (2016). National Health and Nutrition Examination Survey (NHANES): MEC Laboratory Procedures Manual.

[36] Bernert J.T., Harmon T.L., Sosnoff C.S., McGuffey J.E. (2005). Use of continine immunoassay test strips for preclassifying urine samples from smokers and nonsmokers prior to analysis by LC-MS-MS. J. Anal. Toxicol..

[37] Wei B., Feng J., Rehmani I.J., Miller S., McGuffey J.E., Blount B.C., Wang L.A. (2014). High-throughput robotic sample preparation system and HPLC-MS/MS for measuring urinary anatabine, anabasine, nicotine and major nicotine metabolites. Clin. Chim. Acta.

[38] National Health and Nutrition Examination Survey (2019). 2013–2014 Data Documentation, Codebook, and Frequencies: Cotinine, Hydroxycotinine, & Other Nicotine Metabolites and Analogs—Urine (UCOT_H).

[39] National Health and Nutrition Examination Survey (2019). 2015–2016 Data Documentation, Codebook, and Frequencies: Cotinine, Hydroxycotinine, & Other Nicotine Metabolites and Analogs—Urine (UCOT_I).

[40] Tobacco and Volatiles Branch (2014). Tobacco-Specific Nitrosamines.

[41] Xia B., Xia Y., Wong J., Nicodemus K.J., Xu M., Lee J., Guillot T., Li J. (2014). Quantitative analysis of five tobacco-specific N-nitrosamines in urine by liquid chromatography-atmospheric pressure ionization tandem mass spectrometry. Biomed. Chromatogr..

[42] Tobacco and Volatiles Branch (2014). Volatile Organic Compounds (VOCs) Metabolites.

[43] R Core Team (2013). R: A Language and Environment for Statistical Computing.

[44] Merianos A.L., Jandarov R.A., Gordon J.S., Lyons M.S., Mahabee-Gittens E.M. (2020). Child tobacco smoke exposure and healthcare resource utilization patterns. Pediatr. Res..

[45] Merianos A.L., Jandarov R.A., Mahabee-Gittens E.M. (2020). Tobacco smoke exposure, respiratory health, and health care utilization among US adolescents. Chest.

[46] United States Environmental Protection Agency (2025). America’s Children and the Environment: Biomonitoring—Cotinine.

